# Supplementary material for: Multi-Trait Wheat Rhizobacteria from Calcareous Soil with Biocontrol Activity Promote Plant Growth and Mitigate Salinity Stress
Source: Microorganisms. 2021 Jul 26;9(8):1588. doi: 10.3390/microorganisms9081588 (PMC8400701; doi:10.3390/microorganisms9081588)
Supplement: Supplementary file 1 [file microorganisms-09-01588-s001.zip › microorganisms-1293637-SI.pdf]

Supplementary

Multi-trait wheat rhizobacteria from calcareous soil with biocontrol activity promote plant growth and mitigate salinity stress

Anastasia Venieraki [1,*], Styliani N. Chorianopoulou [2], Panagiotis Katinakis [3], Dimitris L. Bouranis [2]

Laboratory of Plant Pathology, Crop Science Department, Agricultural University of Athens, Iera Odos 75, 11855 Athens, Greece

[2] Laboratory of Plant Physiology and MorphologyCrop Science Department, Agricultural University of Athens, Iera Odos 75, 118 55 Athens, Greece s.chorianopoulou@aua.gr (S.N.C.), bouranis@aua.gr (D.L.B.)

[3] Laboratory of General and Agricultural Microbiology, Department of Crop Crop Science Department, Agricultural University of Athens, Iera Odos 75, 118 55 Athens, Greece; katp@aua.gr

*Correspondence: venieraki@aua.gr

Supplementary material

TABLE S1. Biofilm formation (OD_{590})

Bacterial Strain	**OD_{590}**
1.SG.7	1.12 ± 0.095
2.SG.8	0.302 ± 0.02
2.SG.20	0.33 ± 0.08
3.SG.19	0.245 ± 0.06
4.SG.6	0.256 ± 0.09
5.SG.3	0.675 ± 0.13
2.C.19	0.244 ± 0.09
2.C.23	0.289 ± 0.02

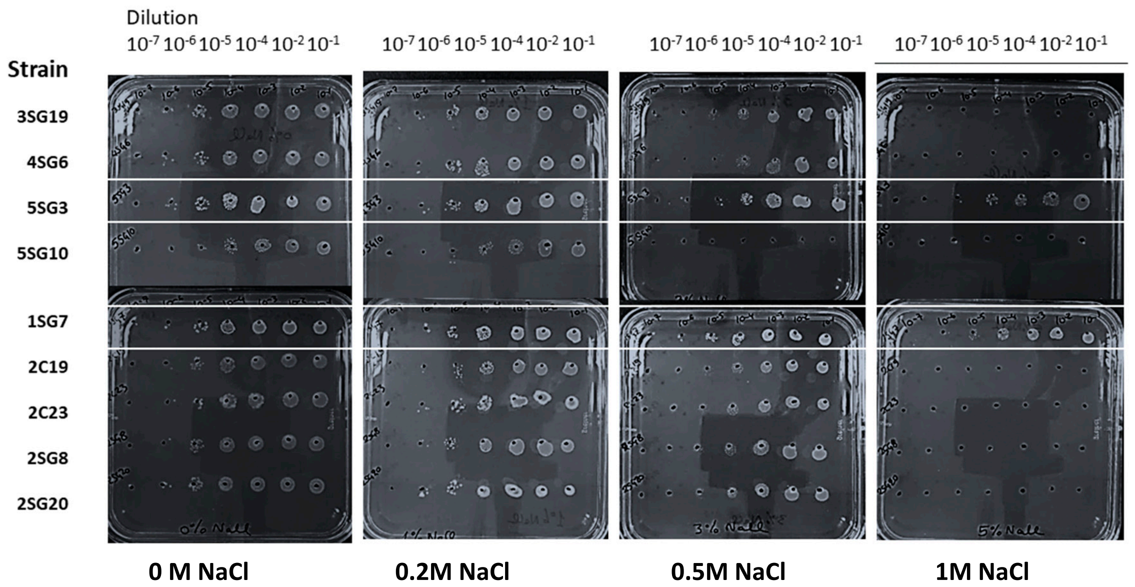

Figure S1. Salinity tolerance of bacterial strains. Eight beneficial arylsulphatase bacterial strains were tested for the ability to grow on NA petri dishes at different NaCl concentrations (0, 0.2M, 0.5M and 1M).

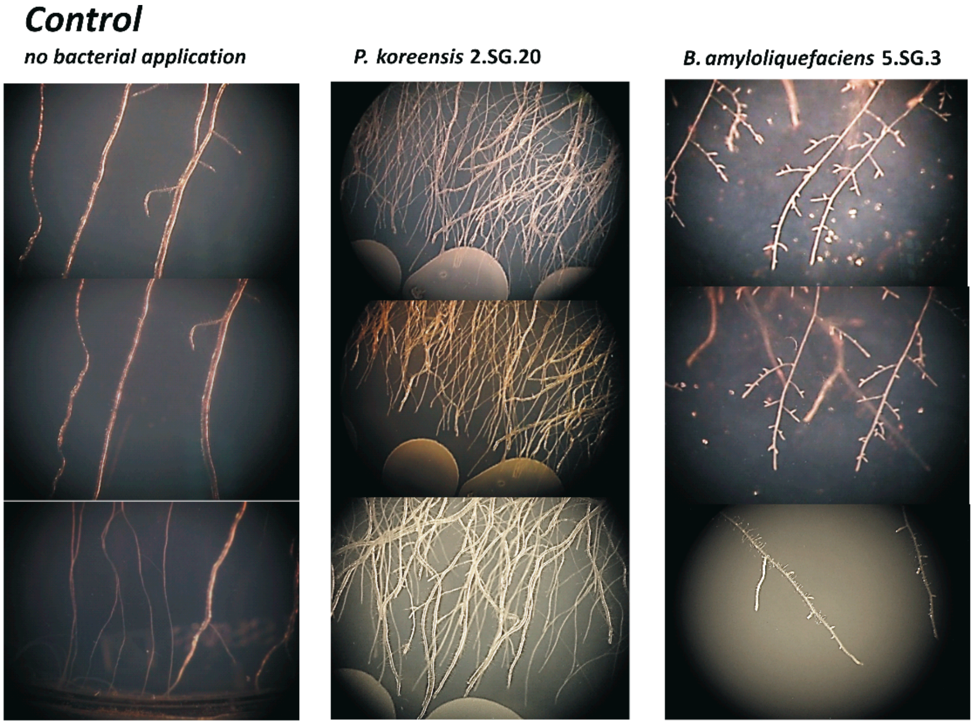

Figure S2. *Arabidopsis thaliana* (Col-0) root architecture under normal conditions. with beneficial bacterial strain (2.SG.20, 5.SG.3) co-inoculation. Stereoscope observation by stereo microscope Leica Wild M3B.

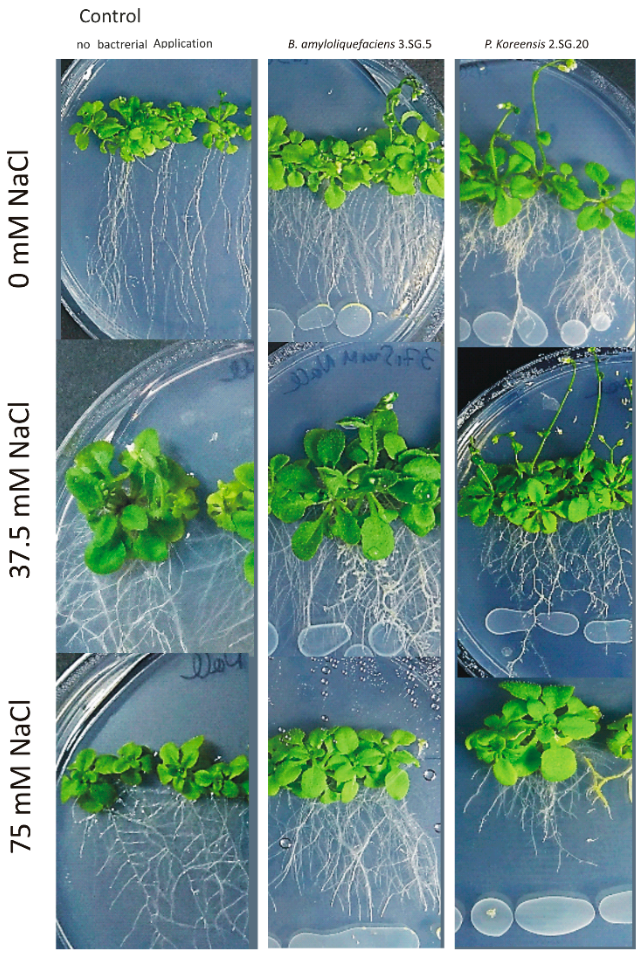

Figure S3. Plant growth and root architecture of *Arabidopsis thaliana* (Col-0) under salinity conditions. Plants were co-inoculated with selected beneficial bacterial strains *in vitro* under salinity stress (0 mM, 37.5 mM, 75 mM NaCl).
